# Patient-Reported Outcome in Dupuytren’s Disease Treated With Fasciectomy, Collagenase or Needle Fasciotomy: A Swedish Registry Study

**DOI:** 10.1016/j.jhsg.2023.06.009

**Published:** 2023-07-19

**Authors:** Madeleine Harryson, Martin Eklund, Marianne Arner, Stephan Wilbrand

**Affiliations:** ∗ Department of Hand Surgery, Örebro University Hospital, Örebro, Sweden; † Department of Medical Epidemiology and Biostatistics, Karolinska Institute, Stockholm Sweden; ‡ Department of Hand Surgery, Stockholm, Sweden; § Department of Clinical Research and Education, Karolinska Institute, Stockholm, Sweden; ‖ Department of Hand Surgery, Uppsala University Hospital, Uppsala, Sweden

**Keywords:** Dupuytren´s contracture, Dupuytren´s disease, Patient-reported outcome, Patient-reported outcome measurement, Swedish National Quality Registry of Hand Surgery (HAKIR)

## Abstract

**Purpose:**

This registry study compares the patient-reported outcomes of 3 treatments for Dupuytren´s disease: open fasciectomy (OF), collagenase injection (CCH) and percutaneous needle fasciotomy (PNF).

**Methods:**

From the Swedish national quality registry for hand surgery (HAKIR) we included 2,585 procedures (in 2,414 patients): 1,200 treatments were OF, 918 CCH, and 467 PNF. The choice between CCH and PNF varied mainly because of regional differences in reimbursement of CCH. We report the results of the validated patient-reported outcome instrument HQ-8. HQ-8 evaluates symptoms in the treated hand and is issued before treatment, 3 and 12 months after treatment and is used for all patients in HAKIR.

**Results:**

At 3-month follow-up, patients treated with CCH or PNF experienced less stiffness, weakness, numbness, tingling and sensitivity to cold. At 12 months**,** the differences among the 3 treatments were smaller, but CCH patients experienced less stiffness and weakness compared to PNF-treated patients.

**Conclusions:**

Most randomized controlled trials have not shown significant differences in recurrence rates or patient-reported outcomes between CCH and PNF, but the number of patients has been limited and no randomized controlled trials have included all 3 treatments. In the present study, we compared registry data on patient-reported outcomes for OF, CCH, and PNF in a real-life clinical setting. Our results confirm that the noninvasive treatments (CCH and PNF) cause less disability than OF and indicate a possible advantage of CCH compared to PNF regarding stiffness and weakness at 1 year after treatment based on patient-reported outcomes. Patient-reported residual symptoms are important to consider when informing patients and selecting treatment for Dupuytren´s disease.

**Type of study/level of evidence:**

Observational registry study III.

The introduction of collagenase treatment (CCH) for Dupuytren’s disease in 2010 increased the scientific interest in this condition. Previously, open fasciectomy (OF) was the gold standard, and the use of percutaneous needle fasciotomy (PNF) was limited mostly to specific countries and non-hand surgeons. Since the introduction of CCH, there has been ongoing debate on the values of the 3 currently available treatment methods. The cost of CCH has been a deciding factor in some countries. Percutaneous needle fasciotomy has seen increasing use, especially after the withdrawal of CCH from the market outside the United States in March 2020.^1^ Randomized controlled trials (RCTs) have not shown significant differences in results between CCH and PNF with respect to recurrence or patient-reported outcomes.^2^^,^^3^ The scientific value of RCTs cannot be overestimated, but the study design makes it difficult to collect large cohorts. In a meta-analysis by Obed et al,^4^ 9 RCTs comparing treatments for Dupuytren’s disease were identified. None of these studies compared all 3 available treatments and the 4 RCTs comparing CCH to PNF included, in total, 345 patients, 50–140 per study. Unlike the other RCTs, Jørgensen et al^5^ found less recurrence using CCH compared to PNF at 3-year follow-up for 77 patients with metacarpophalangeal joint contractures.

To date, to our knowledge, no larger studies have compared patient-reported outcomes of OF, CCH, and PNF or have described perceived side effects, such as stiffness, numbness, tingling, or sensitivity to cold. The present study reports on these aspects using prospectively collected patient-reported outcome data from a large national registry. We hypothesize that patients treated with CCH and PNF experience less stiffness, numbness, tingling, or sensitivity to cold compared to OF-treated patients.

## Materials and Methods

### Study protocol

This observational multicenter registry study was approved by the Swedish Ethical Review Authority, local ethics committee of Stockholm 2016-05-11 (D.nr 2016/158). In accordance with legal requirements, all patients had been informed about the registry and were offered the opportunity to, if so desired. All data for analysis were pseudonymized. The investigators have adhered to the Strengthening of Reporting of Observational Studies in Epidemiology (STROBE) guidelines.

We used prospectively collected data from the national quality registry HAKIR,^6^ which includes information on all performed surgeries (ICD-10 codes and procedural codes) at the hand surgery departments in Sweden, with a coverage of at least 80% of all operations at each unit. Data for all patients treated for Dupuytren’s disease (ICD-10 code M720) from the start of the registry February 9, 2010 to November 14, 2018 were extracted from the HAKIR database. In the registry, patient surveys are issued before treatment, and at 3 and 12 months after surgery to all patients and include the Swedish version of the QuickDASH^7^and the HAKIR-8 (HQ-8).^8^ The HQ-8 is a single-item questionnaire, including 7 questions on symptoms, and 1 on perceived problems in daily activities, all graded 0-100 in 10-point increments (Supplemental Fig. S1, available on the *Journal's* website at www.jhsgo.org).

**Figure 1:**
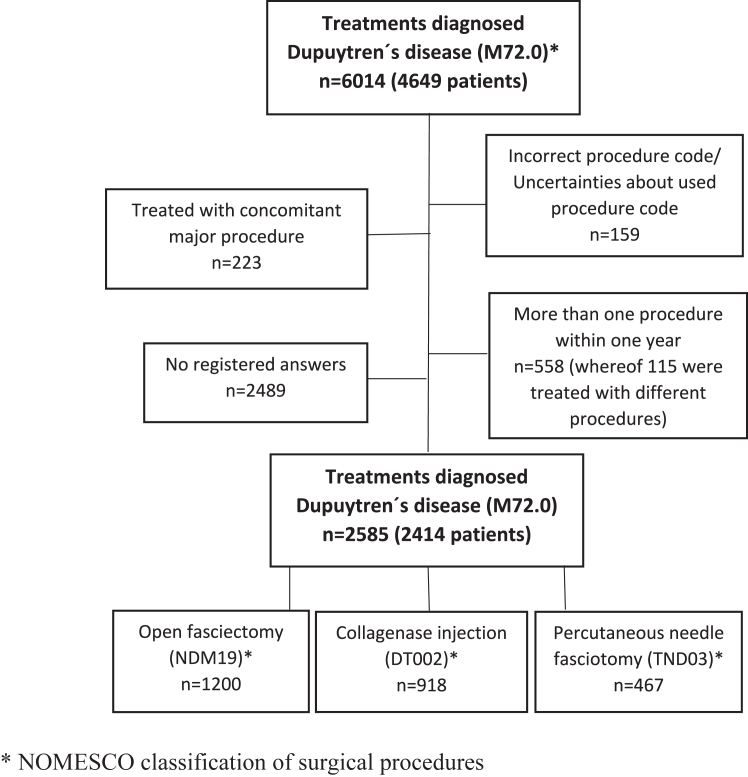
Flowchart on drop-outs.

Treatments were identified by using the procedural codes (NOMESCO classification of surgical procedures)^9^ for OF (NDM19), CCH (DT002) and PNF (TND03). The treatment registrations and the pre- and post-operative patient-reported outcomes were collected and linked together. All patients with at least 1 HQ-8 response were included. Patients treated with concomitant procedures, for example trigger finger release, carpal tunnel release, arthrodesis, and partial or total amputation, were excluded. A number of patients had been treated with more than one procedure within one year. Since the registry only issues one turn of per year, the second procedure was excluded in these patients. Nearly 80% of these patients were treated with the same procedure as previously (Fig. 1).

### Statistical methods

Registry data are observational and contain missing data, leading to fewer cases with full data across the 3 time points (baseline, 3 and 12 months). Therefore, we used 2 approaches to test for differences in HQ-8 score between baseline, and the 3- and 12- month follow-ups:

#### Unadjusted for baseline HQ-8

For this analysis, we used β regression adjusted for age and sex. This model does not adjust for HQ-8 scores at baseline to enable all patients who responded to the questionnaires at different time points to enter the analysis.

#### Adjusted for baseline HQ-8

For this analysis, we used multiple imputation by chained equations to impute HQ-8 scores at each time point (baseline, 3 and 12 months, respectively) using the HQ-8 scores at the other 2 time points as well as age, sex, treatment procedure as covariates. Differences in HQ-8 score between time points then were assessed using β regression, adjusted for age, sex, and HQ-8 score at baseline. We generated 1,000 data sets with imputed data, which were analyzed separately. We then used Rubin’s rules to pool the estimated absolute differences in detection probabilities and standard errors. The imputation and pooling procedure was performed using the R packages mice version 3.9.0 6^10^ and mitools version 2.4 7.^11^

All *P* values and confidence intervals are 2-sided. We report 95% confidence intervals and *P*<.05 was regarded as statistically significant. No correction for multiple tests was performed and confidence intervals for individual contrasts should be interpreted with caution. R statistical software version 4.0.0^12^ was used for all statistical analyses.

## Results

A total of 6,014 treatments of Dupuytren’s disease in 4,649 patients had been registered during the study period; in total 2,414 patients (2,585 procedures) with the diagnosis of Dupuytren’s disease and a major procedure code of either OF, CCH, or PNF were registered in HAKIR and included in the study (Table 1).

**Table 1:** Baseline Characteristics

	Treatment		
	OF, *n* (%)	Collagenase Injections, *n* (%)	PNF, *n* (%)
Age (y)			
Median	67	69	68
IQR	61–72	63–74	62–74
Sex			
Woman, *n* (%)	229 (19)	168 (18)	78 (17)
Man, *n* (%)	971 (81)	750 (82)	389 (83)
Treated hand			
Left, *n* (%)	597 (50)	417 (45)	195 (42)
Right, *n* (%)	603 (50)	500 (55)	259 (55)
Both, *n* (%)	0 (0)	1 (0)	13 (3)

IQR, interquartile range.

**Figure 2:**
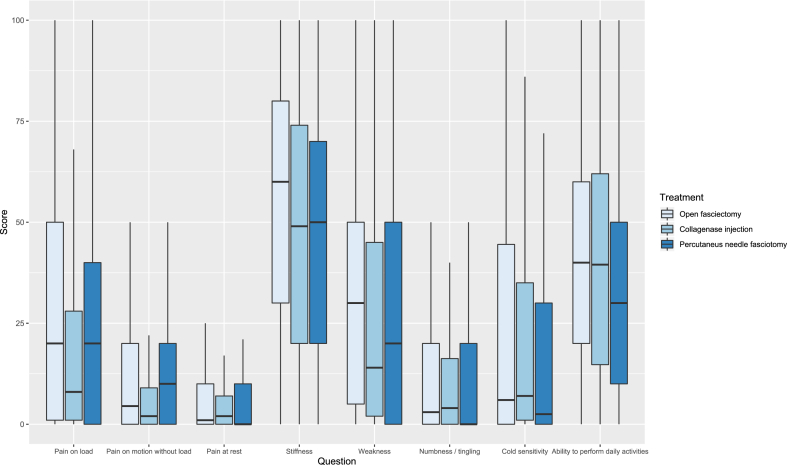
Preoperative patient-reported outcome scores according to treatment and question. The *boxes* indicate interquartile range, *horizontal bold black lines* medians and the *vertical thin lines* show the range between the 25th percentile minus 1.5 interquartile range and the 75th percentile plus 1.5 interquartile range.

**Figure 3:**
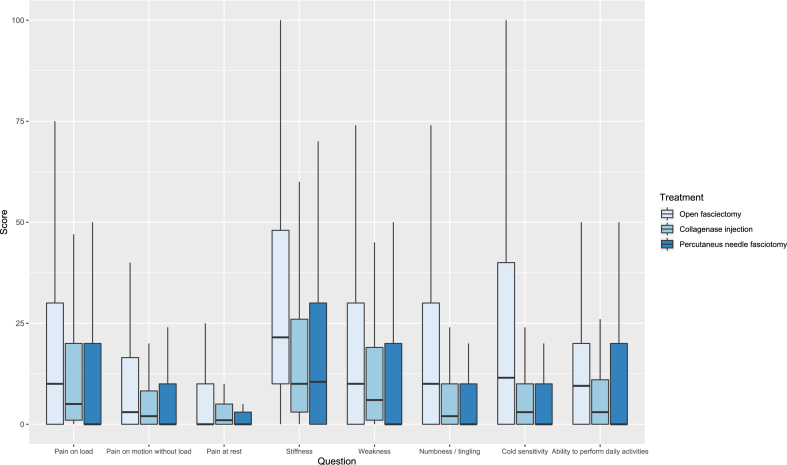
Three months after surgery patient-reported outcome scores according to treatment and question. The *boxes* indicate interquartile range, *horizontal bold black lines* medians and the *vertical thin lines* show the range between the 25th percentile minus 1.5 interquartile range and the 75th percentile plus 1.5 interquartile range.

**Figure 4:**
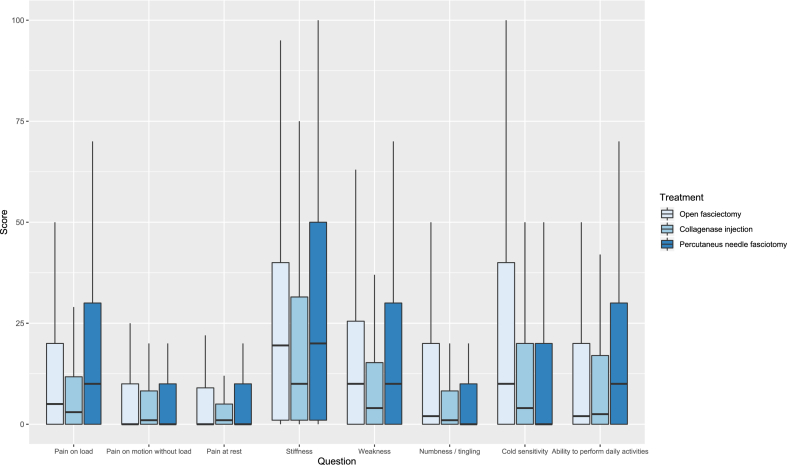
Twelve months after surgery patient-reported outcome scores according to treatment and question. The *boxes* indicate interquartile range, *horizontal bold black lines* medians and the *vertical thin lines* show the range between the 25th percentile minus 1.5 interquartile range and the 75th percentile plus 1.5 interquartile range.

Patient characteristics are shown in Table 1. The preoperative response rate was 56%, at 3 months after surgery 37%, and at 12 months after surgery 52% (Table 2). Nearly all patient-reported outcomes improved from baseline to the 3- and 12-month follow-ups for all 3 treatments. Before surgery, the patients scored most problems with stiffness, weakness, and ability to perform daily activities irrespective of planned treatment (Fig. 2, Table 2). At the 3-month follow-up, the OF group reported more problems with stiffness, weakness, numbness, and tingling, as well as cold sensitivity, compared to the CCH and PNF groups (Fig. 3, Table 2). At the 12-month follow-up, the level of perceived stiffness was stable for OF patients compared to the 3-month follow-up (Fig. 4, Table 2). The PNF patients reported a significantly higher level of stiffness and weakness compared to those with CCH at 12 months (Fig. 4; Tables 2).

**Table 2:** Median and Interquartile Range of Patient-Reported Outcomes at Baseline, and at 3 and 12 Months of Follow-Up

	Baseline			3 mo			12 mo		
	OF	Collagenase	PNF	OF	Collagenase	PNF	OF	Collagenase	PNF
		Injection			Injection			Injection	
Questionnaire responder									
Yes, n (%)	495 (41)	667 (73)	290 (62)	530 (44)	205 (22)	214 (46)	674 (56)	434 (47)	231 (49)
No, n (%)	705 (59)	251 (27)	177 (38)	670 (56)	713 (78)	253 (54)	526 (44)	484 (53)	236 (51)
HQ-8 scores									
Pain on load	20 (1–50)	8 (1–28)	20 (0–40)	10 (0–30)	5 (1–20)	0 (0–20)	5 (0–20)	3 (0–11.75)	10 (0–30)
Pain on motion without load	4.5 (0–20)	2 (0–9)	10 (0–20)	3 (0–16.5)	2 (0–8.25)	0 (0–10)	0 (0–10)	1 (0–8.25)	0 (0–10)
Pain at rest	1 (0–10)	2 (0–7)	0 (0–10)	0 (0–10)	1 (0–5)	0 (0–3)	0 (0–9)	1 (0–5)	0 (0–10)
Stiffness	60 (30–80)	49 (20–74)	50 (20–70)	21.5 (10–48)	10 (3–26)	10.5 (0–30)	19.5 (1–40)	10 (1–31.5)	20 (1–50)
Weakness	30 (5–50)	14 (2–45)	20 (0–50)	10 (0–30)	6 (1–19)	0 (0–20)	10 (0–25.5)	4 (0–15.25)	10 (0–30)
Numbness / tingling	3 (0–20)	4 (0–16.25)	0 (0–20)	10 (0–30)	2 (0–10)	0 (0–10)	2 (0–20)	1 (0–8.25)	0 (0–10)
Cold sensitivity	6 (0–44.5)	7 (1–35)	2.5 (0–30)	11.5 (0–40)	3 (0–10)	0 (0–10)	10 (0–40)	4 (0–20)	0 (0–20)
Ability to perform daily activities	40 (20–60)	39.5 (14.75–62)	30 (10–50)	9.5 (0–20)	3 (0–11)	0 (0–20)	2 (0–20)	2.5 (0–17)	10 (0–30)

When comparing the 3 different treatment methods for all patient-reported variables by follow-up time, unadjusted for baseline levels and excluding imputed data, patients treated with CCH and PNF reported fewer problems with stiffness, weakness, numbness/tingling and cold sensitivity compared to OF patients at the 3-month follow-up (Table 3). At the 12-month follow-up, patients treated with CCH reported fewer problems with numbness/tingling and cold sensitivity than OF patients, PNF patients reported less cold sensitivity than OF patients, and CCH patients reported less stiffness and weakness than PNF patients (Table 3).

**Table 3:** Unadjusted Odds Ratios

	Odds Ratio at 3 mo Follow-Up (95% CI)			Odds ratio at 12 mo follow-up (95% CI)		
	Collagenase Injection vs OF	PNF vs OF	Collagenase Injection vs PNF	Collagenase Injection vs OF	PNF vs OF	Collagenase Injection vs PNF
Pain on load	0.64 (0.39–1)	0.7 (0.44–1.09)	0.91 (0.51–1.6)	0.77 (0.53–1.11)	1.21 (0.8–1.82)	0.63 (0.4–1.01)
Pain on motion without load	0.6 (0.32–1.07)	0.67 (0.36–1.16)	0.9 (0.42–1.89)	0.81 (0.51–1.26)	1.01 (0.59–1.69)	0.8 (0.45–1.45)
Pain at rest	0.56 (0.26–1.09)	0.59 (0.28–1.13)	0.96 (0.39–2.32)	0.77 (0.46–1.27)	0.81 (0.42–1.47)	0.96 (0.49–1.96)
Stiffness	**0.52 (0.34–0.77)**	**0.67 (0.46–0.98)**	0.77 (0.47–1.25)	0.86 (0.65–1.15)	1.37 (0.98–1.91)	**0.63 (0.44–0.91)**
Weakness	**0.63 (0.4–0.99)**	**0.51 (0.31–0.81)**	1.25 (0.7–2.26)	0.74 (0.52–1.04)	1.19 (0.8–1.74)	**0.62 (0.4–0.96)**
Numbness / tingling	**0.48 (0.28–0.78)**	**0.38 (0.22–0.64)**	1.24 (0.64–2.46)	**0.6 (0.4–0.89)**	0.7 (0.43–1.11)	0.86 (0.51–1.49)
Cold sensitivity	**0.47 (0.3–0.74)**	**0.29 (0.17–0.49)**	1.61 (0.86–3.08)	**0.61 (0.44–0.83)**	**0.54 (0.36–0.81)**	1.11 (0.72–1.76)
Ability to perform daily activities	0.63 (0.37–1.03)	0.67 (0.4–1.08)	0.94 (0.5–1.76)	1.05 (0.73–1.5)	1.44 (0.94–2.16)	0.73 (0.47–1.14)

The table shows results unadjusted for baseline HQ-8. The columns show the odds ratios for pairwise comparisons of surgical fasciectomy (NDM19), Collagenase injection (DT002), and PNF (TND03).

Results in bold indicate statistical significance on the 5% level.

When using analyses adjusted for patient-reported outcome levels at baseline, based on multiple imputation (Table 4) the results support the results from the analysis unadjusted for baseline (Table 3). At 3 months, patients treated with CCH experienced less pain on load, pain on motion without load, stiffness, weakness, numbness/tingling, cold sensitivity, and ability to perform daily activities than those treated with OF. The PNF-treated group experienced less problems with stiffness, weakness, numbness/tingling and cold sensitivity than the OF group. At 12 months, CCH-treated patients experienced less numbness/tingling and cold sensitivity than the patients treated with OF, and PNF-treated patients had less cold sensitivity. The CCH-treated patients reported fewer problems with stiffness and weakness than PNF-treated patients.

**Table 4:** Adjusted Odds Ratios

	Odds Ratio at 12 mo Follow-Up (95% CI)			Odds Ratio at 12 mo Follow-Up (95% CI)		
	Collagenase Injection vs OF	PNF vs OF	Collagenase Injection vs PNF	Collagenase Injection vs OF	PNF vs OF	Collagenase Injection vs PNF
Pain on load	**0.67 (0.47–0.95)**	0.72 (0.49–1.04)	1 (0.64–1.56)	0.92 (0.68–1.22)	1.3 (0.92–1.85)	0.72 (0.48–1.08)
Pain on motion without load	**0.62 (0.41–0.95)**	0.66 (0.41–1.07)	0.97 (0.57–1.65)	0.87 (0.58–1.31)	1.11 (0.71–1.74)	0.77 (0.46–1.29)
Pain at rest	0.67 (0.41–1.1)	0.64 (0.37–1.12)	1.01 (0.55–1.84)	0.81 (0.52–1.26)	0.89 (0.54–1.47)	0.87 (0.51–1.47)
Stiffness	**0.58 (0.42–0.8)**	**0.72 (0.54–0.98)**	0.82 (0.57–1.19)	0.94 (0.73–1.2)	1.45 (1.11–1.89)	**0.63 (0.46–0.85)**
Weakness	**0.67 (0.48–0.92)**	**0.56 (0.39–0.81)**	1.15 (0.76–1.73)	0.88 (0.66–1.17)	1.26 (0.88–1.79)	**0.67 (0.47–0.96)**
Numbness / tingling	**0.44 (0.29–0.67)**	**0.39 (0.25–0.62)**	1.13 (0.69–1.85)	**0.68 (0.48–0.96)**	0.81 (0.53–1.24)	0.8 (0.51–1.26)
Cold sensitivity	**0.46 (0.33–0.64)**	**0.39 (0.26–0.58)**	1.2 (0.76–1.89)	**0.6 (0.45–0.8)**	**0.65 (0.46–0.93)**	0.97 (0.62–1.5)
Ability to perform daily activities	**0.65 (0.48–0.89)**	0.79 (0.54–1.15)	0.84 (0.55–1.29)	1.02 (0.76–1.37)	1.62 (1.16–2.27)	0.69 (0.43–1.03)

The table shows results adjusted for baseline HQ-8, using imputation of missing data. The columns show the odds ratios for pairwise comparisons of of surgical fasciectomy (NDM19), collagenase injection (DT002) and PNF (TND03).

Results in bold indicate statistical significance on the 5% level.

## Discussion

In this registry-based study, patients treated with CCH and PNF experienced less numbness/tingling, stiffness, weakness, and cold sensitivity compared to OF patients at 3 months follow-up. At 12-month follow-up, there were fewer significant differences between CCH or PNF and OF, but patients treated with CCH experienced less stiffness and weakness compared to patients treated with PNF.

Advantages of this registry study are the large sample size, the possibility of using data on a nationwide population and involving patients treated by different surgeons in a real-life clinical setting. Another strength of this study is that it involves all types of patients with Dupuytren’s disease, regardless of the severity of the contracture. Of course, the variation in disease severity between patients receiving different treatment modalities should be considered when evaluating the patient-reported outcomes.

Analyzing questionnaire data from registries presents difficulties regarding low response rates, spares details about previous treatments*,* and has the risk of nonresponder bias. However, this risk has been debated. In a large prospective study of hand surgery patients, no significant bias was seen from nonresponders.^13^ This was also found by Ross et al,^14^ analyzing satisfaction after hip and knee arthroplasties between responders and nonresponders. We used multiple imputation by chained equations to impute missing HQ-8 scores at each follow-up time point for nonresponders. Results based on analyses of the imputed data sets (Table 4) were largely similar to results based only on data, supporting that there were no marked differences in HQ-8 scores between nonresponders and responders. In registry studies, it is difficult to control for potential confounders, including selection bias regarding the treatment method. Our data showed that the choice between CCH and PNF mainly depended on where the patient was treated. In some regions almost no CCH treatments were performed, and in other regions almost no PNF. The CCH and PNF procedures have not been registered consistently in HAKIR. Therefore, many more CCH and PNF treatments probably were performed during the study period, but not registered.

A limitation of this study is the lack of information on which finger was treated, the extent of contracture before and after treatment, how many fingers were treated, which joints were contracted, and the sparsely recorded information about whether the treatment was a primary procedure or a reoperation. All these factors could have influenced patient-reported outcomes. Nevertheless, to our knowledge, this is the largest study of patient-reported outcomes for Dupuytren´s disease comparing the 3 treatment modalities, which may compensate for some of the above limitations.

Most previous studies on the treatment of Dupuytren’s disease focus on measurements of remaining contractures^15^, ^16^, ^17^ and no larger studies have compared OF, CCH, and PNF treatments and patient-reported residual problems after treatment. The Unité Rhumatologique des Affections de Main (URAM)^18^ questionnaire has been recommended for evaluating outcomes in Dupuytren´s disease.^17^ The URAM scale provides higher correlation with the Tubiana scale of contracture and patient-assessed disability than, for example, the *Quick*DASH (Disabilities of the Arm, Shoulder, and Hand).^18^ The HAKIR registry is designed to be used for all types of hand surgery and not specifically for Dupuytren´s disease, which is why URAM is not included and not used in this study. The construct validity of the HQ-8 has been investigated and it has been shown to be an important complement to the *Quick*DASH, especially for patients with low levels of pain and disability, such as patients with Dupuytren´s disease.^8^ An additional strength of the HQ-8 compared to the *Quick*DASH is that it is specific to the treated hand, regardless of hand dominance. For these reasons, only data on the HQ-8 questionnaire are used in this study.

When discussing improvement after treatment using patient-reported outcomes, the smallest change in a treatment outcome that a patient would identify as important and not simply statistically significant, has been referred to as the minimal clinically important difference (MCID).^19^ The MCID for *Quick*DASH has been determined,^20^ but has not yet been described for HQ-8. MCID may, vary between different diagnoses using the HQ-8.

In the present study, patients treated with CCH or PNF experienced fewer nerve-related problems, less stiffness, less weakness, and less cold sensitivity during early follow-up compared to surgically-treated subjects. Less differences in perceived sensory disturbance were noted at the 12-month follow-up between OF, CCH or PNF. This is consistent with 2 previous studies with the same follow-up time,^15^^,^^16^ as well as 1 study with 3 years of follow-up.^21^ Our data show significant differences between CCH and PNF regarding experienced stiffness and weakness 1 year after treatment, which possibly could indicate signs of earlier recurrence in the PNF-treated group. In line with this, earlier published studies have suggested that recurrence rate may be higher in patients treated with PNF than in patients treated with collagenase.^5^^,^^22^

In conclusion, we consider it important to include patient perspectives to individualize the care of Dupuytren´s disease, and to understand potential residual problems after treatment. Patients should be informed that less invasive treatment methods in general also give fewer side effects, but that they may come with an increased risk of early recurrence.^23^^,^^24^ Warwick et al^1^ postulated that “different patients have different preferences, while different surgeons have different skills and opinions”.

## Supplementary Data

Online Supplementary Figure 1 HQ-8 questionnaire.
