# Supplementary material for: Patient-Reported Outcome in Dupuytren’s Disease Treated With Fasciectomy, Collagenase or Needle Fasciotomy: A Swedish Registry Study
Source: J Hand Surg Glob Online. 2023 Jul 19;5(6):733–9. doi: 10.1016/j.jhsg.2023.06.009 (PMC10721495; doi:10.1016/j.jhsg.2023.06.009)
Supplement: Online Supplementary Figure 1 — HQ-8 questionnaire. [file mmc1.pdf]

Postoperative questionnaire

HAKIR
HANDKIRURGISKT
KVALITETSREGISTER

☐ **3months postop** ☐ **12 months postop**

Date of birth (social security no) (yyyymmdd-nnnn): ☐☐☐☐☐☐☐☐–☐☐☐☐

Patient questionnaire HQ-8 (arm/hand)

Date (yyyy-mm-dd) ☐☐☐☐-☐☐-☐☐

I am (please indicate your writing hand): ☐ Left handed ☐ Right handed ☐ Ambidextrous

Arm/hand that was operated on: ☐ Left ☐ Right

This questionnaire reports on problems that you have had this past week in the hand/arm that was operated on. Please tick the alternative that best corresponds to any of your problems.

---

1. Pain on load

	0	10	20	30	40	50	60	70	80	90	100	
No problems	☐	☐	☐	☐	☐	☐	☐	☐	☐	☐	☐	Worst problems imaginable

2. Pain on motion without load

	0	10	20	30	40	50	60	70	80	90	100	
No problems	☐	☐	☐	☐	☐	☐	☐	☐	☐	☐	☐	Worst problems imaginable

3. Pain at rest

	0	10	20	30	40	50	60	70	80	90	100	
No problems	☐	☐	☐	☐	☐	☐	☐	☐	☐	☐	☐	Worst problems imaginable

4. Stiffness

	0	10	20	30	40	50	60	70	80	90	100	
No problems	☐	☐	☐	☐	☐	☐	☐	☐	☐	☐	☐	Worst problems imaginable

5. Weakness

	0	10	20	30	40	50	60	70	80	90	100	
No problems	☐	☐	☐	☐	☐	☐	☐	☐	☐	☐	☐	Worst problems imaginable

6. Numbness / tingling in fingers

	0	10	20	30	40	50	60	70	80	90	100	
No problems	☐	☐	☐	☐	☐	☐	☐	☐	☐	☐	☐	Worst problems imaginable

7. Cold Sensitivity (discomfort on exposure to cold)

	0	10	20	30	40	50	60	70	80	90	100	
No problems	☐	☐	☐	☐	☐	☐	☐	☐	☐	☐	☐	Worst problems imaginable

8. Ability to perform daily activities

	0	10	20	30	40	50	60	70	80	90	100	
No problems	☐	☐	☐	☐	☐	☐	☐	☐	☐	☐	☐	Worst problems imaginable

9. How would you rate the result of your operation overall?

	0	10	20	30	40	50	60	70	80	90	100	
Completely satisfied	☐	☐	☐	☐	☐	☐	☐	☐	☐	☐	☐	Totally dissatisfied

10. How would you rate the care that you received at the clinic during your treatment?

	0	10	20	30	40	50	60	70	80	90	100	
Completely satisfied	☐	☐	☐	☐	☐	☐	☐	☐	☐	☐	☐	Totally dissatisfied
